# Digital trade coin: towards a more stable digital currency

**DOI:** 10.1098/rsos.180155

**Published:** 2018-07-18

**Authors:** Alex Lipton, Thomas Hardjono, Alex Pentland

**Affiliations:** MIT Connection Science, Massachusetts Institute of Technology, Cambridge, MA, USA

**Keywords:** blockchain technology, digital currency, electronic cash

## Abstract

We study the evolution of ideas related to creation of asset-backed currencies over the last 200 years and argue that recent developments related to distributed ledger technologies and blockchains give asset-backed currencies a new lease of life. We propose a practical mechanism combining novel technological breakthroughs with well-established hedging techniques for building an asset-backed transactional oriented cryptocurrency, which we call the digital trade coin (DTC). We show that in its mature state, the DTC can serve as a much-needed counterpoint to fiat reserve currencies of today.

## 1. Introduction

This paper describes the concept of the asset-backed *digital trade coins* (DTCs), currently under development at MIT [1]. It outlines an approach to building a consortium of sponsors, who contribute real assets, a narrow bank handling financial transactions involving fiat currencies, and an administrator, who issues the corresponding digital token in exchange for fiat payments and makes fiat payments in exchange for digital tokens. In short, our proposal is to apply distributed ledger technology to give a new lease of life to the old notion of a sound asset-backed currency, and to use this currency as a transactional tool for a large pool of potential users, including small and medium enterprises and individuals. We intend to build a currency, which encourages legitimate commerce, but makes illegal activities difficult. Our contribution should be viewed as a position/vision paper, as at the moment there is no working prototype for the DTC.

We wish to replace physical cash with a supranational digital token, which is insulated from adverse actions by central banks and other parties, due to the fact that it is asset-backed. We believe the DTC is ideally suited as a medium of exchange for groups of smaller nations or supranational organizations, who wish to use it as a counterweight to large reserve currencies.

Supranational currencies have been known for two millennia. For instance, Roman, and later Byzantine and Iranian gold coins were used along the entire Silk Road; Spanish and Austrian silver coins were prevalent medium of exchange in the Age of Sail. Closer to our time, the British Pound was used as reserve currency for the British Empire and, to a lesser degree, the rest of the world; the Dollar and the Pound were used as a reserve currency basket for the world economy in the twentieth century, to which the Euro and the Yen were added in late twentieth century; and now the Yuan might be used along a revived Silk Road.

Today, for the first time ever, there is a possibility of designing a digital currency that combines the best features of both physical cash and digital currencies, including finality of settlement, partial anonymity and usability on the web. This currency is largely immune to policies of central banks that control the worlds' reserve currencies. Such a currency has enormous potential to improve the stability and competitiveness of trading and natural resource producing economies. In the DTC, we propose to develop a trade-oriented asset-backed digital currency, aimed at facilitating international trade and making it as seamless as possible. This currency will be based on a proprietary framework combining the most recent advances in blockchain and distributed ledger technology, cryptography and secure multi-party calculations, together with time-tested methods for preventing double spending. In view of the fact that our framework relies in part on our own research and in part on ideas readily available in public domain, we do not anticipate specific intellectual property right issues. Unlike Bitcoin, it will be fast, scalable and environmentally friendly. It will also be transaction friendly because of its low volatility versus fiat currencies, not to mention cryptocurrencies.

Over the past decade, potential advantages and disadvantages of distributed ledgers or blockchains, have been discussed by numerous researchers (e.g. [2] and references therein). While numerous potential applications of blockchains have been entertained in the literature—including title deeds, post-trade processing, trade finance, rehypothecation and syndicated loans, to mention but a few—the main usage of blockchains has so far been in the general area of payments, more specifically cryptocurrencies.

Worldwide interest in distributed ledgers was ignited by Bitcoin, which is a cryptocurrency protocol operating without a central authority. It was described first in the seminal white paper by Nakamoto [3]. Since then Bitcoin has inspired creation of more than a thousand of other cryptocurrencies, all with various degree of novelty and utility (if any). One of the most promising is Ethereum, which is significantly more versatile than Bitcoin, not least because is supports so-called smart contracts [4]. Another interesting and popular cryptocurrency protocol is Ripple [5]. The Ripple system departs from the Nakamoto consensus approach. It is not truly decentralized because it does not rely on the thousands of anonymous (pseudonymous) mining nodes that form the peer-to-peer network underlying Bitcoin. Instead, the Ripple system uses a small set of nodes that act more like notaries, validating transactions at a higher throughput and much lower cost compared to Bitcoin. Unlike Bitcoin, most entities in the system are known and not anonymous. By their very nature, all of these currencies are native tokens, residing on a blockchain. Their transition from one economic agent to the next is controlled by the set of rules that are inherent or ‘hardwired’ in the blockchain set-up and are needed to maintain the integrity of their blockchain as a whole. However, until now, attempts to build tokens backed by real-world assets—first and foremost, fiat currencies—have been unsuccessful. Yet, until this all important problem is solved, it is virtually impossible to make cryptocurrencies a part of the mainstream financial infrastructure, because otherwise the inherent volatility of cryptocurrencies will severely curtail their usability.

Although potential application of distributed ledgers mentioned earlier, such as post-trade processing and trade finance, are very important, they are technical in nature and lack the revolutionary spirit. However, a distributed ledger can potentially play a truly transformative role and bring a dramatic departure from the past by making *central bank digital currency* (CBDC) and stable cryptocurrencies a reality.

In this work, we propose a stable asset-backed cryptocurrency which we refer to as DTC. It can be viewed as a natural extension of a fiat-backed cryptocurrency called the *utility settlement coin* (USC) [6]. Setting aside operational aspects of gathering and managing collateral assets, we need to design a ledger associated with value transfers. Since, by design, Nakamoto's approach is neither scalable, nor efficient, we need to use a different design. Our analysis indicates that combining blockchain with an earlier approach for issuing electronic cash (e-Cash), developed by Chaum [7,8], seems to be promising. Recall that Chaum introduced a blind signature procedure for converting bank deposits into anonymous cash. On the one hand, Chaum's protocol is much cheaper, faster and more efficient compared to Bitcoin. It also offers an avenue towards true anonymity and unlinkability (as in paper cash), as compared to the weak pseudonymity of Bitcoin. If true anonymity is not desired, there are variations on the Chaum approach on offer, for instance, anonymity for the purchaser but not for seller and so forth. However, on the other hand the basic Chaum model and many of its variants rely on the integrity of the issuing bank. To alleviate this issue, we propose the use of blockchain technology itself to track the relevant transaction parameters, reducing the opportunity for parties to be dishonest. Payments are still direct between users as in Chaum's proposal.

In the DTC, we propose a solution to the stable cryptocurrency problem, which boils down to assembling a pool of *assets*, contributed by *sponsors*, appointing an *administrator*, who will manage the pool and digitizing the ownership rights on this pool. In addition, we build a special-purpose *narrow bank*, which facilitates activities of the administrator. By construction, neither the pool itself nor the supporting bank can fail due to market and liquidity risks. Their operations are streamlined as much as possible to limit operational risks. It is worth noting that operational risks are always present; this statement is true not only for the set-up we are proposing, but for an ordinary cash and bank deposits too, not to mention cryptocurrencies, which are notorious for their operational risk exposures. The narrow bank receives fiat currency submitted by the users, passes it to the administrator and ultimately to sponsors, while the administrator issues digital tokens in return. These tokens will circulate within the group of users in a fast and efficient manner by using distributed ledger mechanism, thus creating native tokens proportionally convertible into the underlying assets at will. Their value is maintained in a relatively narrow band around the value of the underlying asset pool, with lower bound enforced by arbitrage, while the upper bound is enforced by the administrator assisted by sponsors.

The key insight of the paper is that the properly designed DTC can serve as an international reserve currency, remaining stable in the long run and serving as a much-needed counterbalance to fiat currencies issued by individual nations, which can be easily affected by their respective central banks.

The paper is organized as follows. Background on asset-backed currencies is discussed in §2. Design of Bitcoin and Ripple, including their similarities and differences, is outlined in §3. CBDC and closely related USC are discussed in §4. DTC is discussed in §5. Conclusions are drawn in §6.

## 2. Asset-backed currencies

The idea of anchoring value of paper currency in baskets of real assets is old, see, for example [9]. Gold and silver as well as bimetallic standards have been used for centuries to achieve this goal.

Two approaches are common: (i) a redeemable currency backed by a basket of commodities, and (ii) a tabular standard currency indexed to a basket of commodities.

Lowe [10] was the first to explain how to use a tabular standard of value to the price inflation; a similar plan based on a basket of 50 commodities was developed by Scrope [11]. Jevons [12] pushed these ideas (much) further and proposed an indexation scheme based on a basket of a 100 commodities, while Marshall [13] proposed a similar tabular standard.

Inspired by developments during the Great Depression, Graham [14] developed an automatic countercyclical policy based on 100% backing of bank deposits by commodities and goods, while Graham [15] proposed backing the USD with a commodity basket at 60% and gold at 40%. Hayek [16] advocated establishing a universal basket of commodities, which every country would use to back its currency. Roughly at the same time, Keynes [17] designed an international gold-linked multilateral transaction currency, which he called the bancor. Unfortunately, his ideas were discarded by the architects of the Bretton Woods system.

After WWII, interest in commodity-based currencies has been lukewarm. Still, Kaldor [18] proposed a new commodity reserve currency, which he also called bancor. More recently, Zhou [19] proposed a new international reserve currency anchored to a stable commodity basket benchmark.

The choice of the actual asset basket backing DTC is not an easy one. It is partly dictated by the composition of the sponsors' pool and partly by what assets they actually possess and are willing to contribute. For instance, depending on their resources and abilities, sponsors can contribute oil, gold, base metals and agricultural commodities. Given that storage of significant amounts of the above is difficult and costly, it is natural to use collateral, which is in storage already, thus making stored commodities economically productive.

## 3. Existing cryptocurrencies

### 3.1. Bitcoin

Since its first announcement in 2008, Bitcoin [3] has captured the imagination of the public by proposing the first cryptographic electronic currency having no intrinsic value, issued without central authority, and capable of peer-to-peer digital transfers. Anyone can join the Bitcoin ecosystem, which is both a strength and a weakness.

Because it is currently the best-known form of cryptocurrency, it is worth exploring how Bitcoin works. Financial transactions are made directly between users, without the help by designated intermediaries. Transactions are publicly broadcast and recorded in a ‘blockchain ledger’, which can be seen by all participants. Once a transaction is broadcast, the so-called ‘miners’ come into play. They aggregate individual transactions into blocks (currently of about 2000 transactions each), verify them to ensure that there is no double spend by competitively providing *proof of work* (PoW), and receive mining rewards in bitcoins (BTCs). The PoW is based on finding a cryptographic nonce making the hash value of the candidate block of transactions lower than a given threshold. As such, the ‘hash power’ (i.e. hardware and software processing capacity) of a node makes a difference in the likelihood of the node finding the match.

It is assumed (but not proven) that there are sufficiently many honest miners, so that collusion among them (known as 51% attack) is not possible. A transaction is considered to be confirmed if there are at least six new blocks built on the top on the block to which it belongs. The Bitcoin ecosystem is not without very serious issues—it can handle no more than seven transactions per second (versus Visa which can handle more than 20 000 transactions per second), and it consumes enormous amounts of electricity used by miners (by virtue of underlying PoW computation). Thus, the immutability of Bitcoin's blockchain ledger and the prevention of double spending is achieved through mining based on PoW.

**Figure 1.**
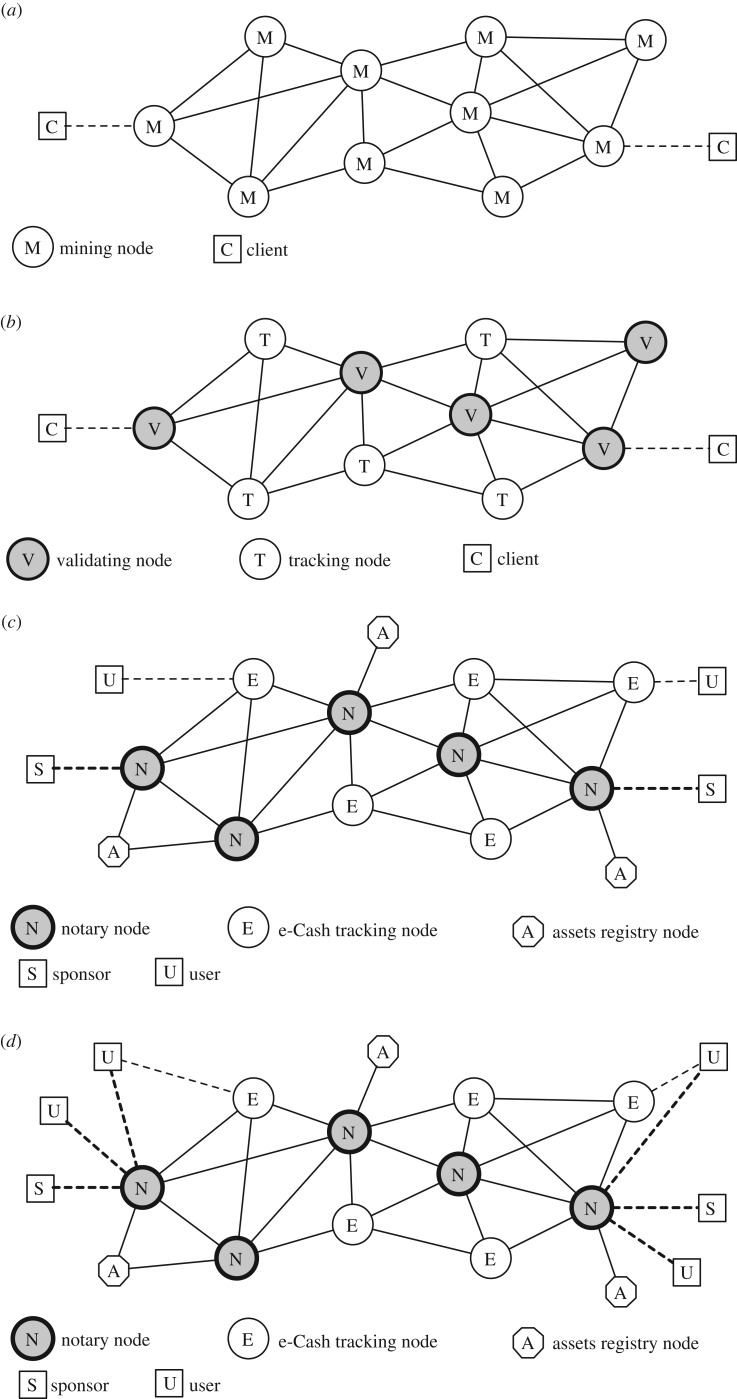
Comparison of different blockchain architectures: (*a*) Bitcoin; (*b*) Ripple; (*c*) DTC for sponsors and (*d*) DTC for sponsors and users.

In view of the above, bitcoins themselves are just unspent transactions outputs of a long chain of transactions, which can be traced all the way back to the time when it was minted, either to the very first ‘genesis’ block, or as part of a ‘coinbase’ transaction included in a block by a successful miner. Bitcoin architecture is shown in figure 1*a*.

Since Bitcoin's inception in 2009, its price has gone up several orders of magnitude, making it the darling of speculators across the globe. However, a word of caution is in order. Since Bitcoin has no value, it can have any price, hence one should not be surprised if its price falls dramatically. Other than for speculative purposes, Bitcoin's uses are rather limited, because its price versus the US dollar and other fiat currencies is extremely volatile, which prevents it from becoming a medium of transaction. In addition, in spite of claims to the opposite, Bitcoin transaction costs are very high and growing.

Although Bitcoin may not be the disruptive force as its supporters are claiming, its underpinning distributed ledger technology has a clear potential to transform the financial ecosystem as a whole.

### 3.2. Ripple

Ripple is a money transfer protocol; ripple is the underlying native currency. It is completely different from Bitcoin. For starters, ripples are preminted, while bitcoins are mined. In fact, Ripple is not decentralized at all. The stated purpose of the protocol is to facilitate fiat currency transfers among participating banks. However, due to the fact that there is a native token, Ripple can also be used along the line of Bitcoin. Details of how Ripple works are given in various Ripple promotional materials including their white paper [5].

The main ingredients of the Ripple ecosystem can be summarized as follows: (i) Servers, which maintain the ledger; (ii) Clients, who can initiate transactions; (iii) Proposers, which can be any Server and (iv) the Unique Nodes List (UNL), indicating parties which can be trusted by the participants in the protocol.

The life cycle of a single transaction consists of several steps. First, a transaction is created and signed by an account owner. Second, this transaction is submitted to the network; if it is badly formed, this transaction may be rejected immediately; otherwise, it is provisionally included in the ledger. Validating nodes propose new ledger. Transmitting nodes broadcast it to the network. Consensus is achieved by voting of the validators. The result of a successful consensus round is a validated ledger. If a consensus round fails, the consensus process repeats until it succeeds. The validated ledger includes the transaction and its effects on the ledger state.

Ripple consensus assumptions are (A1) every non-faulty Server makes decision in finite time; (A2) all non-faulty Servers arrive at the same decision and (A3) *both* true and false decision regarding a given transaction are possible.

Ripple Protocol Consensus Algorithm (RPCA) works in rounds: (A) initially, every Server compiles a list of valid candidate transactions; (B) each Server amalgamates all candidates coming from its UNL and votes on their veracity; (C) transactions passing the minimum threshold are passed to the next round; (D) the final round requires 80% agreement. In general, RPCA works well; however, it can fail provided that validating nodes form cliques which cannot agree with each other. Ripple architecture is shown in figure 1*b*.

## 4. Central bank digital currency and utility settlement coin

### 4.1. Central bank digital currency

Could and should central banks issue *central bank digital currency*? Recently, a previously academic question of feasibility and desirability of CBDC came to the fore (e.g. [20–31]). By issuing CBDC, states can abandon physical cash in favour of its electronic equivalent and replace a large chunk of government debt with it. The impact on society at large will be huge [32]. CBDC can obviate the need for fractional banking and dramatically improve the stability of the financial system as a whole. On the other hand, the ability of the banking sector to create money ‘out of thin air’ by making loans will be significantly curtailed and transferred to central banks. It is clear that developments in this direction are inevitable, but their timing and magnitude are uncertain.

Interest in CBDC has been ignited by two unrelated factors—the introduction of Bitcoin and a persistence of negative interest rates in some developed countries. In Medieval Europe, negative interests existed in the form of demurrage for centuries. Recall that demurrage is a tax on monetary wealth. In principle, demurrage encourages spending money, rather than hoarding it, thus accelerating economic activity. The idea of demurrage was reborn shortly after WWI in the form of scrip money, which requires paying of periodic tax to stay in circulation. Scrip money was proposed by the German-Argentinian entrepreneur and economist Gesell [33], whose idea was restated by Irving Fisher during the great depression [34]. Demurrage was thought to be a suitable replacement for mild inflation. Since in the modern economy demurrage is hard to orchestrate due to the presence of paper currency, its conversion into the electronic form is necessary for making seriously negative rates a reality [26].

Currently, there are three approaches to creating CBDC on a large scale:

(A) Economic agents, from enterprises to private individuals, can be given accounts with central banks. However, in this case, central banks would have to execute know your customer (KYC) and anti-money laundering (AML) functions, tasks which they are not equipped to perform. Besides, under duress, rational economic agents might abandon their commercial bank accounts and move their funds to central bank accounts, thus massively destabilizing the entire financial system.

(B) Inspired by Bitcoin [3], CBDC can be issued as a token on an unpermissioned distributed ledger, whose integrity is maintained by designated notaries receiving payments for their services (e.g. [35]). Given that notary efforts do not require mining and hence are significantly cheaper and faster than that of Bitcoin miners, this construct is scalable and can satisfy needs of the whole economy. Users are pseudo-anonymous, since they are represented by their public keys. Since at any moment there is an immutable record showing the balance of every public key, it is possible to de-anonymize transactions by using various inversion techniques applied to their recorded transactions [36], thus maintaining AML requirements.

(C) A central bank can follow the Chaumian scheme [7,8], and issue numbered and blind signed currency units onto a distributed ledger, whose trust is maintained either by designated notaries or by the bank itself. In this case, it would have to rely on commercial banks, directly or indirectly, for satisfying the KYC/AML requirements.

To summarize, by using modern technology, it is possible to abolish paper currency and introduce CBDC. On the positive side, CBDC can be used to alleviate some of the societal ills and eliminate costs of handling physical cash, which are of order of 1% of the country's GDP. It can help the unbanked to participate in the digital economy, thus positively affecting the society at large. On the negative side, it can give central authorities too much power over the economy and privacy, which can potentially be misused.

While CBDC is absolutely stable with respect to the underlying fiat currency, it does not make the fiat currency stable in itself. For that we need a carefully constructed DTC.

### 4.2. Utility settlement coin

The CBDC is technically possible but politically complicated. Hence several alternatives have been proposed. One promising venue is USC, which is developed by a consortium of banks and a fintech start-up called Clearmatics.^1^ Initially, USC can be an internal token for a consortium of participating banks. These coins have to be fully collateralized by electronic cash balances of these banks, which are held by the Central Bank itself. Eventually, these coins can be circulated among a larger group of participants. However, in this case, issuance of USCs has to be outsourced to a narrow bank, which can perform the all-important KYC and AML functions.

Recall that a narrow bank has assets, which include solely marketable low-risk securities and central bank cash in an amount exceeding its deposit base as per the regulatory prescribed capital cushion (e.g. [37] among many others). As a result, such a bank is impervious against credit and liquidity shocks. However, as any other firm, it can be affected by operational failures, including fraud, computer hacking, inability to solve the KYC/AML problem, etc. These failures can be minimized, but not eliminated, by virtue of using proper modern technology. Accordingly, narrow bank deposits would be as close to the fiat currency, as technically possible.^2^ Ideally, one narrow bank per fiat currency is required. Further details are given in [6].

The USC is helpful from a technical perspective, but it does not solve issues of monetary policy. We wish to address this issue by building a counterweight for fiat currencies by backing the DTC by a pool of real assets.

### 4.3. Survivability of central bank digital currency and utility settlement coin

The idea that a blockchain system can withstand a concerted attack simply because it consists of physically distributed nodes is an untested and unproven proposition. The possible types of attacks to a blockchain system have been discussed elsewhere and consist of a broad spectrum. These range from classic network-level attacks (e.g. network partitions, distributed denial of service) to more sophisticated attacks targeting the particular blockchain-specific constructs (e.g. consensus implementations), to targeting specific implementations of mining nodes or notaries (code vulnerabilities, viruses, etc.). An attack on a blockchain system may not need to cripple it entirely—a degradation in its overall service quality (e.g. slower transaction throughput) may be sufficient to disincline users to use the system.

The notion of *interoperability* across blockchain systems is an important one in the light of survivability [38]. Internet was able to expand and allowed *autonomous systems* (i.e. routing domains) to interconnect with one another due to good design principles. The design philosophy of the Internet is based on three fundamental goals, namely (i) network survivability (Internet communications must continue despite loss of networks or gateways); (ii) variety of service types (Internet must support multiple types of communications service) and (iii) variety of networks (Internet must accommodate a variety of networks). We believe the same fundamental goals must be adopted for the current development of blockchain technology—and more specifically they must drive the technological selection for the implementations of the DTC architecture.

## 5. Digital trade coin design principles and requirements

### 5.1. Digital trade coin: motivations

Bitcoin and Ripple protocols can be used as a prototype for a distributed ledger-based cryptocurrency more suitable for transactional financial purposes. Several issues, some technical and some economical, have to be addressed before this goal can be achieved:

(A) the KYC problem has to be formulated and articulated and a suitable framework for solving it has to be designed;
(B) an AML mechanism has to be developed;
(C) a highly efficient method for maintaining consensus on the ledger, with the industrial strength transactions per second (TpS) capabilities, has to be built;
(D) a transparent and economically meaningful system for issuing new DTCs and retiring the existing ones has to be implemented;
(E) and, most importantly, a satisfactory mechanism for making DTC a stable cryptocurrency has to be designed.

Although public ledgers are not truly anonymous, but rather pseudonymous, it is difficult to use them in the KYC/AML compliant fashion. Accordingly, the DTC ledger has to be made semi-private (but probably not private) in order to solve the KYC/AML problem. At the same time, a right balance has to be struck between privacy and accountability, so that excessive restrictions should not impede the flow of legitimate commerce.

In order to achieve the level of speed and efficiency we are aspiring for, including TpS of order several thousand, the Ripple-style consensus protocol has to be used. Following Ripple's approach, we choose a group of notaries, who are known in advance and properly licensed. These notaries are responsible for performing ledger updates and maintaining its integrity by ensuring Byzantine fault tolerance (e.g. [39,40]). For their services notaries are paid a small fee, say a percentage of the transaction amount they approve, which is naturally denominated in DTC, so that their commercial interests are aligned with their functions. If notaries stay inactive, or systematically approve invalid transactions, they are financially penalized. In each round, validators create their own versions of the ledger and propose these to the rest of the group. Several rounds of voting take place until a super-majority candidate ledger is selected. This approach is similar in spirit to the well-known Paxos algorithm. In order to increase TpS number, we use the idea of sharding and assign individual notaries to particular sets of addresses. In this set-up, a quorum verifies its own shard, while the full ledger is assembled out of the corresponding shards.

The DTC architecture recognizes that there are two or three types of application-level transactions commonly found in many blockchain implementation. The first is the one-party recording of assets to the ledger. Logically, the DTC represents this on an *assets ledger*. The second type is the two-party transferral transaction, exemplified by the transferral of coins from one party to another. The DTC captures these logically on the *coins ledger*. The third type of transaction is the off-chain transferral of value (i.e. e-Cash) in a privacy-preserving manner. Here the goal is to allow a limited amount of coin-backed anonymous e-Cash to be transferred from one user to another, following the classic Chaum approach. Relevant parameters of the e-Cash flow are recorded on the DTC *tracking ledger* in order to reduce the opportunity of fraud by entities involved in the e-Cash flows.

This design decision of recognizing the three types of application-level transactions provides the broadest flexibility for the DTC architecture to be tailored for specific use cases, and for different implementations of the three ledgers to be chosen according to the requirements of the use case.

### 5.2. Creation and annihilation of digital trade coin

For now, we shall consider the asset pool and its associated narrow bank as given, and describe the creation and annihilation mechanisms for the DTC. New coins are injected in the distributed ledger by virtue of the following mechanism. During the initial stage, participants who wish to acquire a freshly minted DTC have to proceed in several steps. First, they have to have a conventional fiat account, which can be held either directly with the narrow bank or with their commercial bank. Second, they have to open an initially empty wallet ready to accept DTCs. Third, participants transfer the desired amount of fiat currency to the narrow bank. Fourth, the narrow bank transfers these funds to sponsors who, in turn, release some of the DTCs created when the asset pool is built to the pool administrator. Fifth, the administrator transfers the corresponding DTCs from its public key address to the public key address provided by the participant. Thus, in effect, participant becomes a shareholder in the pool administrator. Subsequently, participants can acquire DTCs from other participants in exchange for goods and services, so that a newly born DTC starts its journey from one address represented by a public key to the next, until it is annihilated by a participant sending it to the administrator in exchange for cash. When a participant in the ledger wishes to receive fiat currency for their DTC, they transfer DTCs from their public key to the public key of the administrator, who, in turn, sells an appropriate proportion of the assets, deposits proceeds with the associated narrow bank, which, in turn, credits fiat currency either to the account on its own ledger or to a designated account in a different bank. The corresponding DTCs are destroyed by sending them to the ‘terminal’ public key without a private key.

As a result, the administrator is in possession of real assets, sponsors with fiat currency, general public with DTCs, which can always be converted into fiat at the current market price.

### 5.3. Mechanisms of stabilization of digital trade coin

Finally, the value of the DTC is kept relatively stable by virtue of the independent actions of participants and the administrator. If the value of a DTC goes below the value of the fraction of the asset pool it represents, which we call its intrinsic value, then rational economic agents will turn it back to the administrator in exchange for cash. If, on the other hand, the market value starts to deviate upward compared to the intrinsic, then, after a certain threshold is breached, the sponsors will contribute more assets to the pool, which can come from their own sources or be purchased on the open market, in exchange for DTCs, which they will sell on the open market, thus pushing the market price of DTCs down. These two complementary mechanisms can keep the market price of the DTC in a bank around the market price of the underlying basket.

More precisely, the price *P*_DTC_ of DTC will be close to (but not exactly at) the market price of the corresponding asset pool, *P*_M_. Indeed, if *P*_DTC_ falls significantly below *P*_M_, economic agents will put DTC back to the administrator, who will have to sell a fraction of the pool's assets for cash and pass the proceeds to these agents. If *P*_DTC_ increases significantly above *P*_M_, sponsors will supply more assets to the administrator, who will issue additional DTC and pass them to sponsors, who will sell them for cash, just pushing the price down. This mechanism ensures that |*P*_DTC_ − *P*_M_|/*P*_M_≪1, a very desirable feature, especially compared with conventional cryptocurrencies, habitually exhibiting extreme volatility. At the same time, outright manipulation by central banks is not possible either.

Note that the notion of economic agents (e.g. sponsors with assets) is distinct from system entities (e.g. notaries) in the DTC architecture (see below).

### 5.4. System design principles

In order for DTC to be a stable and durable digital currency that can store value as well as provide utility, there are a number of principles driving its architecture. The DTC architecture seeks to be a ‘blueprint’ that allows the DTC to be implementable for various use cases. Some use cases which have been identified are (i) a reserve digital currency shared by a number of geopolitically diverse small countries, as a means to provide local financial stability; (ii) a digital currency operating by a narrow bank that can provide relative stability during financially volatile periods. A number of system design principles are as follows:

— *Unambiguous identifiability and ownership*. Assets (represented digitally), coins and e-Cash must be uniquely identifiable, and have unambiguous ownership at any given time. A corollary of true ownership is that these must be transferable (portable) by its owner.
— *Visibility into shared state*. Entities in the ecosystem should have visibility into the state of the DTC system and network, and have equal access to such information. More specifically, this means visibility into the assets which back the issuance of coins, and visibility into the circulation of coins and e-Cash.
— *Mechanisms implementing monetary policies*. In order for the DTC ecosystem to operate according to the desired community behaviour, there must be technical mechanisms that allow agreed policies to be carried out in the system as a whole. Such mechanisms can be controlled centrally (e.g. single entity), controlled in a group-oriented manner (e.g. consensus of entities) or a combination of both (e.g. leader election protocols).
— *Unambiguous authenticable identification of entities*. Entities and system components must be unambiguously identifiable and authenticable. This means that human participants, user-driven devices and network machines/nodes must each be authentically identifiable.
— *Correct, accurate and unhindered system-wide reporting*. System components that implement DTC must each be unhindered in the reporting of its internal state. Furthermore, there must be ways to validate reported state, so that misbehaviour can be detected and acted upon. Such misbehaviours can be the result of human or system error, degradation in system components over time (hardware and software) or the result of active or passive compromises (i.e. attacks).

The above system design principles borrow from a number of key design principles underlying Internet [38]. The need for unambiguous ownership of an asset is an obvious one. The DTC seeks to use standard object identification solutions (e.g. GUID standard) for digital assets. The legal ownership of assets is a construct that is external to the DTC system, and as such must be established prior to assets being introduced by its legal owner (e.g. sponsor) into a given DTC deployment.

The principle of visibility is driven by the need for entities in a DTC implementation to have equal access to data, and is implemented through the assets ledger and coins ledger. The consortium administration (see below) must have full visibility into all operational aspects of a given DTC implementation. Certain DTC implementations may restrict visibility of parts of the systems (e.g. assets ledger) to entities that have ‘skin in the game’ (e.g. sponsors who have actual assets on the DTC assets ledger).

A key aspect of the success of a DTC implementation is the ability of the consortium to carry out monetary policies and other governance rules in the system as a whole. Technical mechanism can be implemented as ‘hooks’ or control-points through which policy decisions are executed. For example, a DTC implementation may require that each sponsor have assets (in the assets ledger) above a given threshold (i.e. reserve ratio) at all times. The actual value of the threshold should be dynamically adjustable according to the consortium-agreed policies and be carried out by the consortium administration as the appointed authority. In this case, the consortium administration can transmit a special ‘policy implementation’ transaction (to the assets ledger and coins ledger) setting the new threshold value. Notaries observe such policy decisions by declining an asset-to-coin conversion transaction from a sponsor if it causes the sponsor's asset reserves to dip below the new threshold value.

Key to the operation of a DTC implementation is the ability of entities to identify and authenticate each other. We believe this is closely related to the principle of system-wide reporting. Some DTC implementation may choose to deploy advanced cryptographic techniques that provide anonymity and untraceability of entities. However, such features must still satisfy the principle of unambiguous identifiably and mutual authentication.

### 5.5. Sponsors, consortium and users

**Figure 2.**
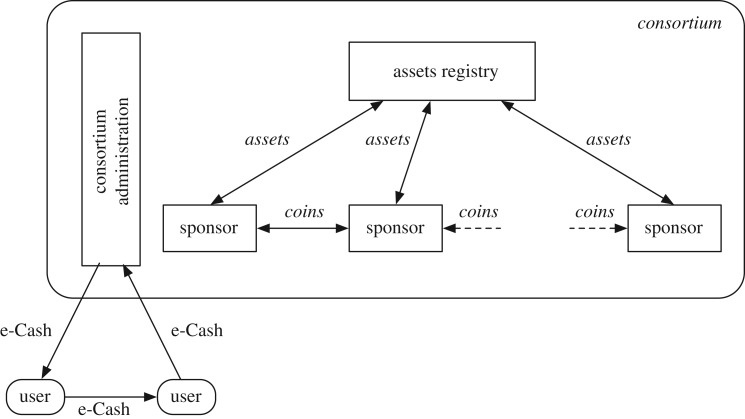
Tradecoin entities.

There are a number of active (human-driven) entities in the DTC ecosystem (figure 2):

— *Sponsor*. A sponsor is an entity who supplies assets to the DTC ecosystem in return for coins. The community of sponsors forms a consortium (see below) tasked with the various management aspects of coins and e-Cash in the ecosystem.
— *Consortium*. A community of sponsors forms a consortium, operating under an agreed governance model that specifies the legal, business and technical operational rules of members of the consortium. In essence, the consortium is a network of sponsors who are participating in the DTC ecosystem.
  Additionally, a *Consortium Administration* carries out the monetary policies of the membership of the consortium. The consortium administration is legally empowered by the consortium membership to implement (centralized) control over certain system functions.
— *Users*. A user is an entity that obtains e-Cash from the consortium for the purpose of payments for goods and services from other users.

DTC architecture is shown in figure 1*c,d*.

### 5.6. Logical functions

The DTC architecture logically separates functions into those pertaining to assets, coins and e-Cash. Here we use the term *ledger* generically without calling out specific realizations, to allow focus on logical functions that meet the system design principles stated above.

Specific technical implementations of the ledger may include a distributed database system, a peer-to-peer network of nodes, a fully distributed blockchain system, or even an append-only single database system.

**Figure 3.**
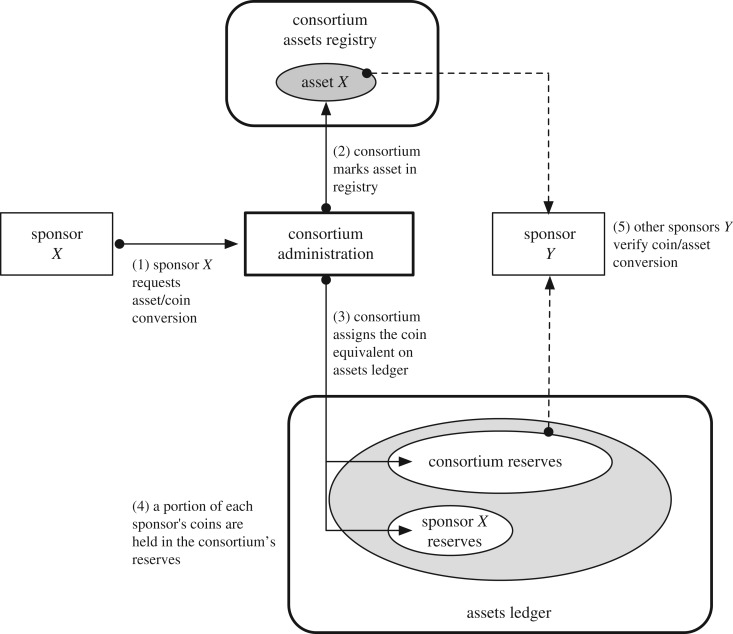
Converting assets to coins.

**Figure 4.**
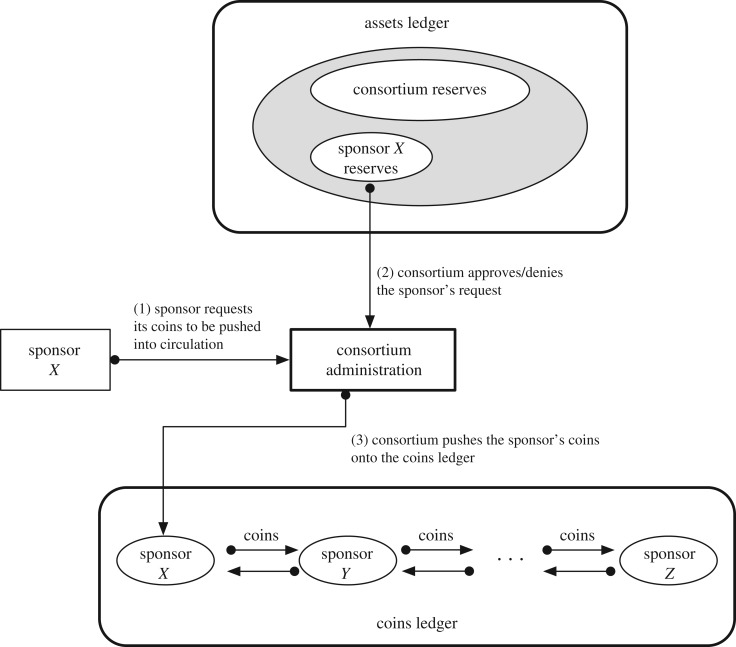
Tradecoin coins ledger.

— *Assets management*. Visibility into the assets which sponsors contribute in exchange for coins represents a foundational requirement in DTC. DTC employs an *assets registry* and an *assets ledger* (figure 3). The registry records verified real-world assets that is associated to a sponsor who forwards that asset to the consortium.
  The assets ledger captures the binding between real-world assets (put forward by a sponsor) and the amount of coins equivalent to (proportional to) those assets. The assets ledger also records the proportion of coins that are in the consortium's reserves and those that are in a sponsor's reserve. These coin-equivalents are considered to be non-circulation.
— *Coin circulation*. Allowing sponsors to exchange (i.e. sell or lend) with each other their asset-backed coins represents a cornerstone of DTC. The *coins ledger* records the coin movements and transactions in the DTC ecosystem (figure 4). The coins ledger is used by sponsors and the consortium administration. Sponsors exchange or ‘trade’ coins with each other on this ledger.
— *e-Cash circulation*. Providing stable digital currency to users also represents a cornerstone of DTC. The e-Cash *tracking ledger* records the movement of e-Cash (i.e. cryptographic keys and parameters) between users.

Each of the three ledgers in DTC are independent, but are connected in the sense that transaction in one ledger may refer to (point to) recorded transactions in other ledgers. This independence of ledgers is important not only from the perspective of technological choice (i.e. adoption of new ledger technologies), but also crucial to the operational resilience of the system as a whole.

An example of the connection of the ledgers is the ‘pushing’ (or pulling) of coins into (out of) circulation by a sponsor following the policies of a given DTC implementation. When a sponsor seeks to have its assets (on the assets ledger) be converted to coins and for the resulting coins to be accessible by the sponsor on the coins ledger, the sponsor must transmit a push-transaction. This results in a transaction occurring on the assets ledger and a corresponding transaction occurring on the coins ledger. These two transactions—albeit on different ledgers—are related in that one refers to (i.e. carries a hash of) another. In the push case, the transaction on the coins ledger points to a completed transaction on the assets ledger.

### 5.7. Converting assets to coins

The purpose of the assets ledger together with the assets registry is to satisfy the design principles with regards to the conversion of real-world assets into its coin equivalent.

A key requirement here is the validation of the legal ownership of assets as claimed by a given sponsor. The sponsor must provide legal evidence in such a way that a digital representation of the evidence can be captured and presented within the assets ledger.

Examples of such evidence include a paper certificate and its digital representation that has been digitally signed by the issuer using legally acceptable digital-signature technology (e.g. Digital Signature Act of 2000). For example, a digital version of a gold certificate (e.g. unallocated gold) could be signed by an authority and presented by a sponsor as evidence. It is the responsibility of the consortium administration to validate the evidence.

### 5.8. Coins for sponsors

The medium for sponsors to exchange coins with each other is the *coins ledger*. The notion here is that coins to be bought, lent and returned among sponsors are on the ledger, providing transparency and visibility into the trading behaviour of all sponsors in the DTC network.

Prior to having access to coins on this ledger, a sponsor must explicitly request the consortium to ‘push’ the sponsor's coins from the assets ledger (from the sponsor's reserves) into circulation on the coins ledger.

The consortium administration must respond to this request in an explicit manner (request granted, denied or postponed) on the assets ledger. A request that is granted is followed by the consortium administration transferring coins from its account on the coins ledger to the sponsor's account on the same ledger.

This explicit request–response paradigm is a manifestation of the mechanism to implement monetary policies (as mentioned previously in §5.4). It is a ‘hook’ into the system in which the consortium administration—as the representative of the community of sponsors—enforces policies agreed to by the community.

A simple example of a monetary policy decision is the *reserve ratio* that must be met by each sponsor on the assets ledger. A sponsor that exhausts its reserves on the assets ledger, thereby violating the policy of sponsors maintaining a minimum reserve, should not be granted a request to push further coins into circulation on to the coins ledger.

A symmetric operation to pushing coins to the coins ledger is that of ‘pulling’ coins from circulation. This may occur when a sponsor wishes to enlarge its reserves on the assets ledger by moving coins from the coins ledger to the assets ledger.

### 5.9. e-Cash for users

A third important aspect of DTC is its use of e-Cash for users in the ecosystem. In general, a *user* is distinguished from a sponsor in that a user does not possess assets in the consortium. The user obtains e-Cash in exchange for fiat currencies that are acceptable by the consortium. The goal of the user is to use a convenient and low-cost (zero-cost) e-Cash payment method, one that is stable on a day-to-day basis and which can store value over a reasonably long period of time.

In DTC, the entity that issues and redeems is the consortium itself. This ensures that the stability of e-Cash is directly related to the stability of coins and assets in the consortium, all three of which are under the monetary control of the consortium as a community.

The consortium as the issuer of e-Cash to a user enacts monetary policies that govern how much e-Cash a user can request specifically at any one time. More generally, the consortium can govern how much e-Cash is permitted to be in circulation at any given moment in time, as a function of the total assets at the consortium.

**Figure 5.**
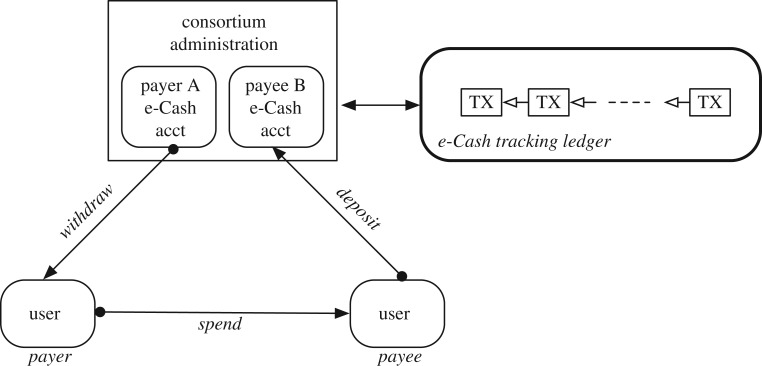
Tradecoin e-Cash tracking ledger.

The e-Cash *tracking ledger* (figure 5) is used for the purposes of fraud-prevention, and does not hinder the flow of e-Cash as understood in the classical Chaum sense. Electronic cash—first defined in 1981 by David Chaum [7]—employs a direct transfer paradigm between users, involving the delivery of a number of cryptographic parameters. Different variants or schemes of e-Cash (e.g. [7,8,41,42]) deploy differing cryptographic parameters. As such, the purpose of the tracking ledger is to record the cryptographic hash of these parameters with the goal of reducing fraud and error, and providing post-event audit and accountability.

## 6. Conclusion

In this paper, we have discussed conceptual underpinnings and technical approaches to building DTCs. We have shown that DTCs have several decisive advantages compared with more established cryptocurrencies, such as Bitcoin and Ripple. In addition to being convenient transactional cryptocurrencies for the Internet era, DTCs can serve as important counterbalance to fiat currencies, and, when fully developed, can play the role of a supranational currency facilitating international commerce and allowing groups of small countries to create their own viable currencies.

As Dr Zhou Xiaochan, Governor of the Peoples Bank of China stated in [19], ‘The desirable goal of reforming the international monetary system, therefore, is to create an international reserve currency that is independent from individual nations and is able to remain stable in the long run, thus removing the inherent deficiencies caused by using credit-based national currencies.’ We believe that DTC has the potential to provide such an international reserve currency for the following reasons: (A) DTC has real value because its price is pinned to a representative basket of commodities; (B) the price of the DTC versus a fiat currency has very low volatility compared with other cryptocurrencies; (C) as a result, DTC can be used as a transaction currency (think of a mortgage taken in DTC in a country which is naturally aligned with some of the major constituent commodities); (D) DTC can also be used as a unit of account and a store of value (as much as gold or oil, say, can).

To conclude, DTC can serve as a much-needed counterpoint for fiat currencies.

## Notes

1 The lead author is a member of their advisory board.

2 As always, Shakespeare put it best: Neither a borrower nor a lender be, for loan oft loses both itself and friend, and borrowing dulls the edges of husbandry. Hamlet, Act 1, Scene 3.

## Data accessibility

This article has no additional data.
